# Metformin protects lens epithelial cells against senescence in a naturally aged mouse model

**DOI:** 10.1038/s41420-021-00800-w

**Published:** 2022-01-10

**Authors:** Mengmeng Chen, Yushan Fu, Xu Wang, Ruitong Wu, Dongmei Su, Nan Zhou, Yanhua Qi

**Affiliations:** 1 Department of Ophthalmology, The Second Affiliated Hospital of Harbin Medical University, 150001 Harbin, China; 2 Department of Ophthalmology, Xixi Hospital of Hangzhou, 310023 Hangzhou, China; 3 Department of Genetics, National Research Institute for Family Planning, Health Department, 100000 Beijing, China

**Keywords:** Senescence, Drug discovery

## Abstract

The senescence of lens epithelial cells (LECs) is a major factor leading to age-related cataract (ARC). ARC results in visual impairment and severe vision loss in elderly patients. However, the specific mechanism of ARC remains unclear, and there are no effective therapeutic agents to halt the formation of ARC. This study aimed to assess the underlying mechanism of the formation of ARC and investigate the potential anti-ageing effect of metformin (MET) on ARC. Male C57BL/6 mice were divided into three groups: the control group having young mice (3 months old, *n* = 40), the naturally aged group (aged 20 months, *n* = 60) and the MET group (MET, 20 months, *n* = 60). Mice in the control and the naturally aged groups were fed a standard purified mouse diet ad libitum and water, whereas those in the MET group were fed chows supplemented with 0.1% MET for 10 months. The transparency of the lens and age-associated proteins p21 and p53 were analysed in the LECs of these three groups. Furthermore, we determined the expressions of the adenosine monophosphate (AMP)-activated protein kinase (AMPK) pathway and the effect of MET on this pathway in LECs during the ageing process of ARC. In addition, the relationship between autophagy and the senescence of LECs and the role of MET in the autophagy of LECs during the ageing process of ARC were examined. Our results indicated that age-related inactivation of the AMPK pathway and impairment of autophagy might contribute to the senescence of LECs and the occurrence of ARC. More importantly, these results demonstrated that MET effectively alleviated the senescence of LECs and the formation of ARC probably via inactivation of the AMPK pathway and augmentation of autophagy. These findings revealed that MET can be exploited as a potentially useful drug for ARC prevention. Our study will help in enlightening the development of innovative strategies for the clinical treatment of ARC.

## Introduction

Lens epithelial cells (LECs) form a monolayer of cells lining the anterior capsule of the lens and extend to the equatorial lens bow [1, 2]. The normal construction and function of LECs are essential for the maintenance of the transparency and metabolic homeostasis of the entire lens [3]. Increasing evidence indicates that the senescence of LECs may cause modification, denaturation and aggregation of lens proteins including enzymes and crystallins, thereby ultimately leading to lens opacification or even cataract [3–5]. Age-related cataract (ARC) remains the leading cause of visual impairment and blindness, which severely affects the quality of life of older individuals [6]. Currently, there are no effective strategies for the prevention of progression of ARC. Therefore, it is necessary to develop a pharmacological intervention that can improve the transparency of the lens and delay ARC progression.

Over the past few decades, scientists have achieved remarkable progress in improving health and extending lifespan using several genetic, dietary and pharmacological interventions [7]. Metformin (MET) is one of the extensively studied anti-ageing agents. Substantial studies suggest that MET can alleviate senescence and extend a healthy lifespan in various animal model systems. MET can not only prolong the lifespan of *Caenorhabditis elegans* but also enhance its health and vitality [8]. MET can reduce the age-related and oxidative stress-related accumulation of DNA damage in *Drosophila* midgut stem cells to extend the lifespan [9]. Previous studies also indicate that chronic low-dose MET administration improves the health and lifespan of mice [10]. Moreover, emerging evidence indicates that when patients with diabetes are treated with MET, they manifest survival benefits, even when compared with controls who do not have diabetes [11].

In addition to the lifespan-promoting activity of MET in various model organisms, MET targets multiple cellular signalling pathways to ameliorate age-related diseases. MET has been shown to modulate autophagy mainly via adenosine monophosphate (AMP)-activated protein kinase (AMPK) activation and prevent neurodegeneration and neuroinflammation to play a neuroprotective role in Parkinson’s disease [12]. Similarly, MET ameliorates age-related changes in the liver sinusoidal endothelial cell via the AMPK and endothelial nitric oxide pathways [13]. Moreover, chronic AMPK activation by MET prevents cardiomyopathy by upregulating autophagy in OVE26 mice with diabetes [14]. In addition, MET alleviates age-associated cardiovascular deterioration by enhancing autophagy and caloric restriction [15]. Furthermore, MET treatment can inhibit age-associated atherosclerosis in ApoE^−/−^ mice and rabbits induced by a high-fat diet, significantly preventing atherosclerotic plaque calcification, reducing high-sensitivity C-reactive protein levels and repressing the activation of the nuclear factor kappa B (NF-κB) pathway in blood vessels [16–18].

Based on these observations, the present study attempted to explore whether MET can delay ARC ageing and the underlying molecular mechanism. Our findings are as follows: (i) chronic low-dose MET treatment delayed the senescence of LECs in naturally aged mice, thereby inhibiting lens opacity; (ii) the AMPK pathway was inactivated, and autophagic flux was impaired in senescent LECs; (iii) chronic low-dose MET treatment delayed the senescence of LECs in naturally aged mice by activating the AMPK pathway and enhancing autophagy. Our findings substantiate the putative anti-ageing role of MET and provide sufficient evidence that MET may prove to be a possible therapeutic option for ARC.

## Results

### Senescence of LECs was associated with ARC in naturally aged mice

**Fig. 1:**
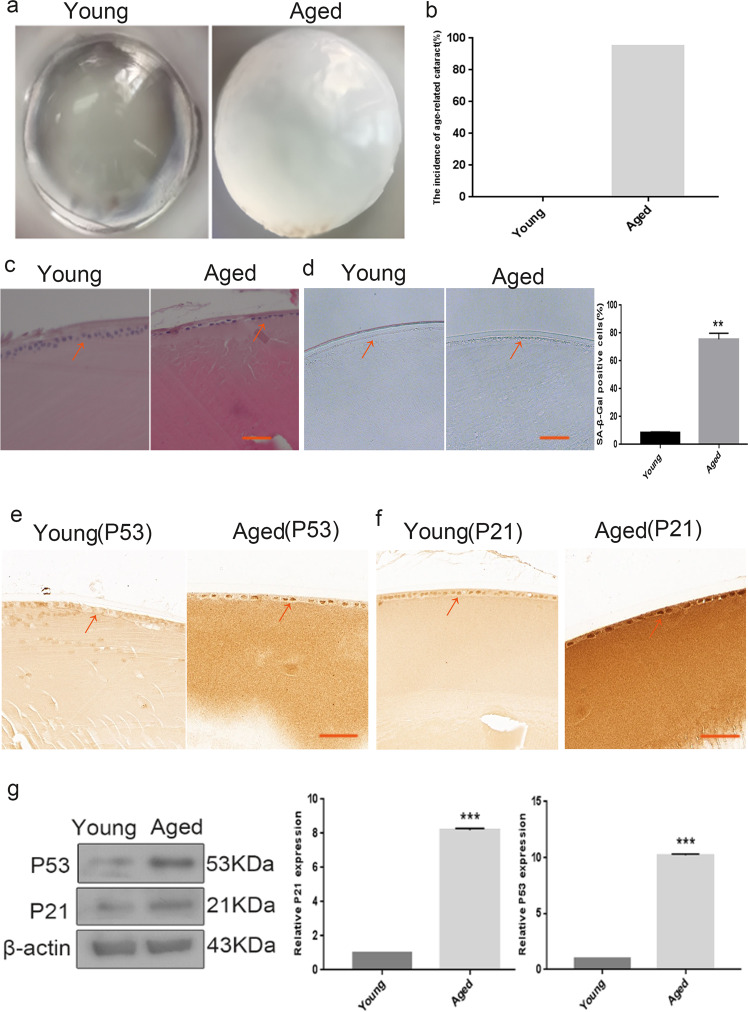
Senescence of lens epithelial cells was associated with the ARC in naturally aged mice. **a** Representative images of morphological observations of the lens in the Young (left) and the Aged (right). **b** The cataract incidence in the Young (left) and the Aged (right). **c** The lens of the Young (left) and the Aged (right) were analysed by H&E staining. **d** Representative images of SA-β-Gal staining of the Young (left) and the Aged (right) and the percentages of SA-β-Gal-positive cells in the Young and the Aged. **e** Representative images from IHC assays against P53 in the Young (left) and the Aged (right). **f** Representative images from IHC assays against P21 in the Young (left) and the Aged (right). **g** Western blot analysis of P53, P21 and β-actin in the Young and the Aged. Data were shown as mean ± SD and are representative of 3 independent experiments. **p < 0.01; ***p < 0.001 compared to the Young. The bar represents 20 μm.

Multiple potential mechanisms of ARC, including the effect of ageing, have been extensively investigated. However, the association between the senescence of LECs and ARC in vivo remains unclear. We hypothesised that the senescence of LECs may result in ARC in vivo. Therefore, we constructed a mouse model of ARC. Our results revealed that the transparency of the lens in naturally aged mice (older mice) was significantly decreased as compared with that in the control group (young mice) (Fig. 1a). As shown in Fig. 1b, the incidence of cataract in the naturally aged group was 95.23%. H&E staining assay indicated that the LECs in the control group were round in shape and arranged regularly, and the density of the normal LECs was relatively high, whereas the LECs of the naturally aged mice were flat in shape and arranged unevenly, and the density of senescent LECs was markedly decreased (Fig. 1c). In addition, SA-β-Gal staining showed that the ratio of senescent LECs was elevated in naturally aged mice (Fig. 1d). Furthermore, we analysed the protein expression of senescence-associated markers via IHC staining and western blot analysis. IHC staining revealed that the expression of p21 and p53 was increased in the LECs of naturally aged mice as compared with young mice (Fig. 1e, f). Similarly, we further discovered that the protein expression of p21 and p53 was higher in the lens capsules of naturally aged mice than in the lens capsules of young mice (Fig. 1g). These results suggested that the senescence of LECs was associated with ARC in naturally aged mice.

### Chronic low-dose MET administration improved the transparency of the lens and alleviated the senescence of LECs

Before we tested the effect of metformin on ARC, we determined whether the chronic low-dose MET administration is safe for mice. After MET treatment for 10 months, 28 mice (18 in the naturally aged group and 10 in the MET group) died of natural causes. We found that alive mice in both groups were healthy, and there was no significant difference in body weights and organ weights between both groups (Table 1). Therefore, these results indicated that the dosage of metformin used in the study was safe for the animals and could be used for subsequent experiments.

**Table 1:** The organ index of mice (data are presented as mean ± S.E.M).

Group	Body	Len	Brain	Heart	Kidney
	Weight (g)	Weight (mg/g)	Weight (mg/g)	Weight (mg/g)	Weight (mg/g)
Young	29.72 ± 1.66	0.08 ± 0.02	18.62 ± 1.49	7.33 ± 0.91	7.45 ± 1.23
Aged	32.98 ± 1.93	0.09 ± 0.02	18.43 ± 1.17	7.27 ± 1.06	7.69 ± 2.23
MET	31.21 ± 2.81	0.09 ± 0.02	18.99 ± 1.63	7.15 ± 0.89	7.56 ± 1.88

**Fig. 2:**
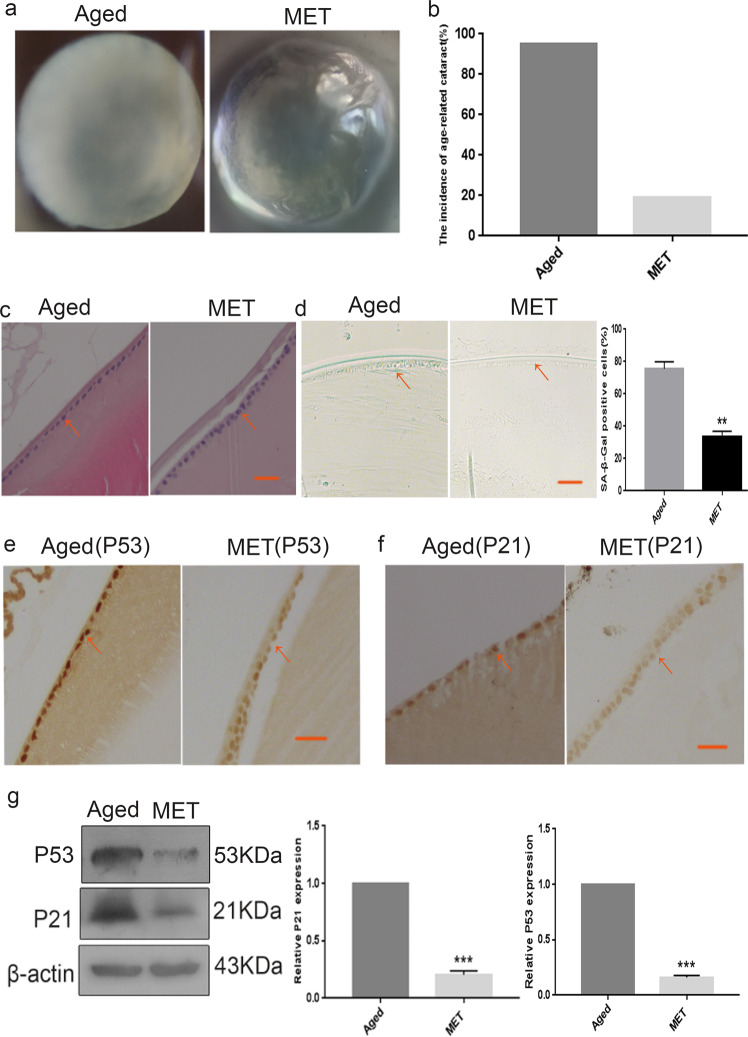
Chronic low-dose MET administration served the transparency of lens and alleviated the senescence of lens epithelial cells. **a** Representative images of morphological observations of lens in the Aged (left) and the MET (right). **b** The cataract incidence in the Aged (left) and the MET (right). **c** The lens of the Aged (left) and the MET (right) were analysed by H&E staining. **d** Representative images of SA-β-Gal staining of the of the Aged (left) and the MET (right) and the percentages of SA-β-Gal-positive cells in the Aged and the MET. **e** Representative images from IHC assays against P53 in the Aged (left) and the MET (right). **f** Representative images from IHC assays against P21 in the Aged (left) and the MET (right). **g** Western blot analysis of P53, P21 and β-actin in the Aged (left) and the MET (right). Data were shown as mean ± SD and are representative of 3 independent experiments. **p < 0.01; ***p < 0.001 compared to the Aged. The bar represents 20 μm.

Subsequently, chronic low-dose MET was administered to assess whether MET could inhibit lens opacity and relieve age-related senescence of LECs in naturally aged mice. As expected, the transparency of the lens in the MET group was greatly improved as compared with the transparency of the lens in the naturally aged group (Fig. 2a). In addition, morphological observations confirmed that the lens of the MET group showed a reduction in opacity. The incidence of cataract in the naturally aged group was 95.23%, whereas that in the MET group was 19.00% (Fig. 2b). H&E staining assay revealed that the density of LECs in the MET group was notably higher than that in the naturally aged mice (Fig. 2c). Moreover, the LECs of the MET group were relatively round in shape as compared with those of the naturally aged group (Fig. 2c). SA-β-Gal staining showed that the ratio of senescent LECs was significantly decreased in the MET group as compared with that in the naturally aged group (Fig. 2d). IHC analysis revealed that the expression of p21 and p53 was decreased more in the LECs of the MET group than in the naturally aged group (Fig. 2e, f). Similarly, western blot analysis revealed that the protein expression of p21 and p53 was remarkably decreased in the lens capsules of the MET group as compared with those of the naturally aged group (Fig. 2g). These results signified that chronic low-dose MET administration could improve the transparency of the lens and alleviate the senescence of LECs.

### Chronic low-dose MET administration attenuated the senescence of LECs in naturally aged mice via AMPK activation

**Fig. 3:**
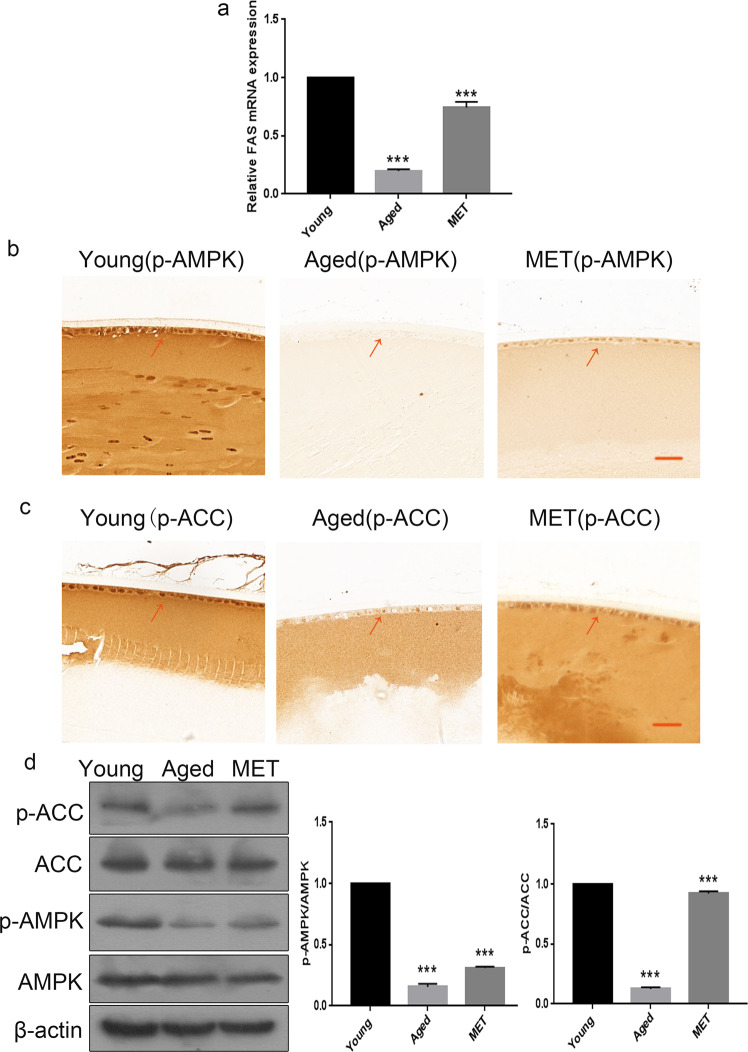
Chronic low-dose MET administration attenuated the senescence of lens epithelial cells in naturally aged mice via AMPK activation. **a** Relative fold-changes in the mRNA levels of the genes encoding FAS as determined by qRT-PCR. **b** Representative images from IHC assays against p-AMPK in the Young, the Aged and the MET. **c** Representative images from IHC assays against p-ACC in the in the Young, the Aged and the MET. **d** Western blot analysis of AMPK, p-AMPK, ACC, p-ACC and β-actin in the Young, the Aged and the MET. Data were shown as mean ± SD and are representative of 3 independent experiments. **p < 0.01; ***p < 0.001 compared to the Young. The bar represents 20 μm.

An overwhelming amount of data supports the conclusion that activating AMPK is sufficient to extend lifespan in model organisms, increasing its appeal as a longevity target [19, 20]. Considering this theory, we assessed the expression of the AMPK pathway in LECs of mice. The relative FAS mRNA level was reduced in the naturally aged group and increased in the MET group (Fig. 3a). As shown in Fig. 3b, c, the expression of phosphorylated AMPKα (Thr172) and phosphorylated ACC (Ser79) was reduced in the LECs of naturally aged mice as compared with those of young mice. However, the expression of phosphorylated AMPKα (Thr172) and phosphorylated ACC (Ser79) was elevated in the LECs of the MET group as compared with those of the naturally aged group. In addition, western blot analysis revealed that the protein expression of phosphorylated AMPKα (Thr172) and phosphorylated ACC (Ser79) was significantly downregulated in the lens capsules of the naturally aged, and the protein expression levels of phosphorylated AMPKα (Thr172) and phosphorylated ACC (Ser79) were increased in the lens capsules of the MET group (Fig. 3d). These data revealed that the AMPK pathway was inactivated in the LECs of naturally aged mice. Furthermore, chronic low-dose MET administration attenuated the senescence of LECs in naturally aged mice via AMPK activation.

### Chronic low-dose MET administration restored the autophagic flux to alleviate the senescence of LECs in naturally aged mice

**Fig. 4:**
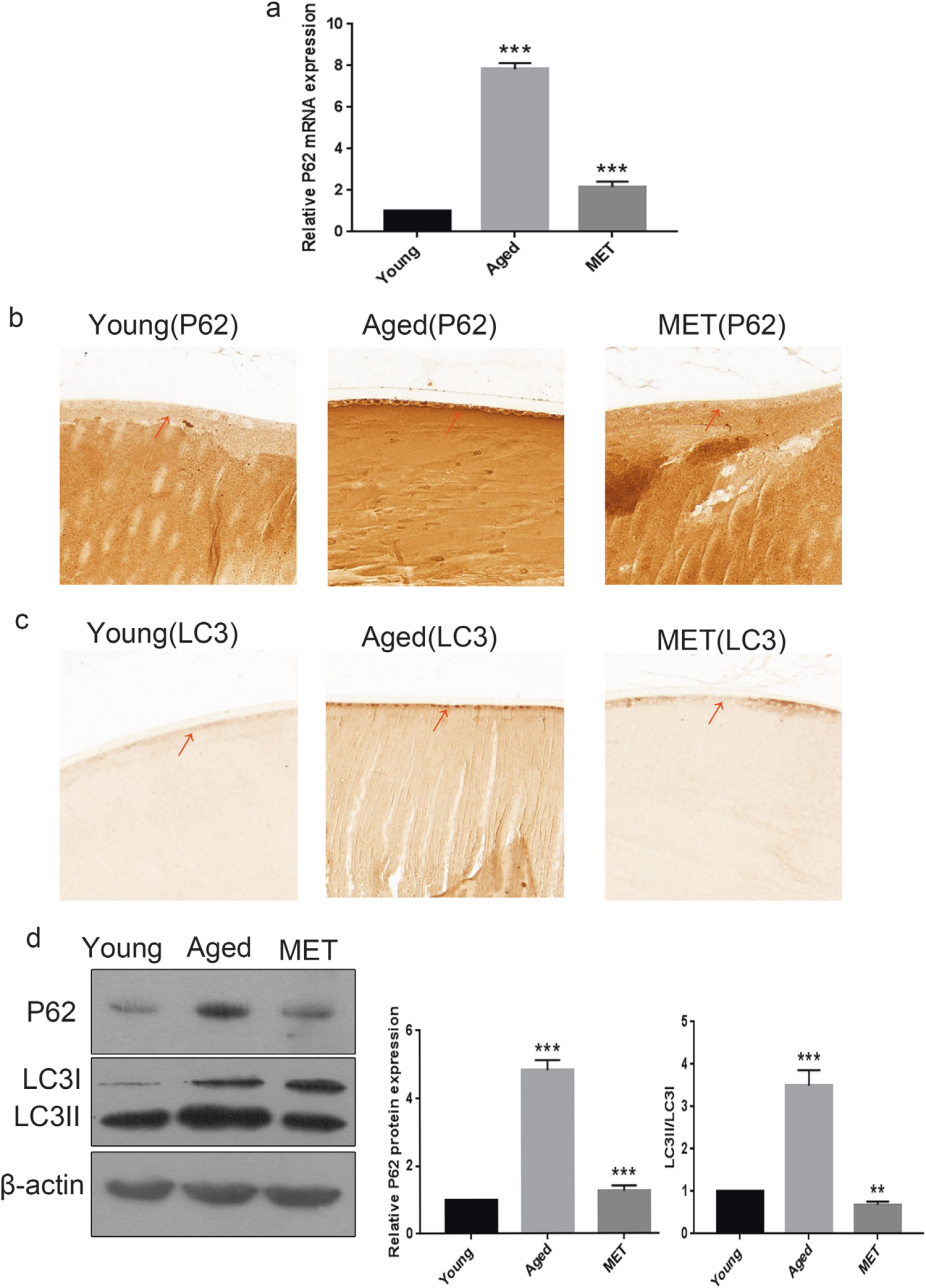
Chronic low-dose MET administration restored the autophagic flux to attenuate the senescence of lens epithelial cells in naturally aged mice. **a** Relative fold-changes in the mRNA levels of the genes encoding P62 as determined by qRT-PCR. **b** Representative images from IHC assays against P62 in the Young, the Aged and the MET. **c** Representative images from IHC assays against LC3 in the Young, the Aged and the MET. **d** Western blot analysis of AMPK, p-AMPK, ACC, p-ACC and β-actin in the Young, the Aged and the MET. Data were shown as mean ± SD and are representative of 3 independent experiments. **p < 0.01; ***p < 0.001 compared to the Aged. The bar represents 20 μm.

Previous studies have indicated that autophagy may play an important role in combating the adverse effects of ageing [21, 22]. To further analyse the molecular and cellular changes in the lens after MET treatment, we determined the expression of LC3 and p62 because they are widely accepted as biomarkers of the levels of autophagy. As demonstrated in Fig. 4a, the relative p62 mRNA expression level was increased significantly in the naturally aged group as compared with that of the control group; however, the relative mRNA expression level of p62 was substantially downregulated in the MET group as compared with that in the naturally aged group. In addition, IHC staining revealed that the levels of microtubule-associated protein 1 light chain 3 (LC3-II/I) and p62 were increased in the LECs of naturally aged mice. Furthermore, our results illustrated that the levels of LC3-II/I and p62 were remarkably decreased in the LECs of the MET group (Fig. 4b, c). The protein levels of LC3-II and p62 were markedly increased in the lens capsules of the naturally aged group (Fig. 4d). Altogether, we speculated that the autophagic flux was impaired in the LECs of naturally aged mice and could be restored by chronic low-dose MET administration to alleviate the senescence of LECs in naturally aged mice.

## Discussion

Untreated cataracts are a leading cause of blindness worldwide that are characterised by opacification of the crystalline lens [23]. Epidemiological studies have shown that age is the dominant risk factor for cataracts, known as ARC, and the only treatment is surgical removal [24]. Previous studies have shown that the senescence of LECs is the main cause of ARC [25]. To date, there have been no effective therapeutic agents to inhibit the cloudiness of lens and attenuate the senescence of LECs without undesirable side effects. The focus of the present study was to assess the molecular biological basis for the senescence of LECs and the mechanism of protecting normal LECs against senescence. Our results revealed that inactivation of AMPK and impairment of autophagy were associated with the senescence of LECs and ARC. Furthermore, we found that MET might protect LECs against senescence and inhibit the cloudiness of the lens by activating AMPK and enhancing autophagy.

Senescence is a fundamental biological process accompanied by a general decline in tissue function. As the lifespan increases, age-related dysfunctions such as ARC become serious health issues [26]. Senescence of LECs has been reported to be involved in the occurrence and development of ARC [5]. Our results revealed that the LECs of the naturally aged mice were flat in shape and arranged unevenly, and the density of LECs decreased markedly (Fig. 1c, d). In addition, the expression of ageing-associated genes (p21 and p53) was upregulated in the LECs of the naturally aged mice (Fig. 1e, f, g). Our results implied that the senescence of LECs was associated with the occurrence and development of ARC. Therefore, an accurate understanding of the molecular mechanisms underlying the senescence of LECs may be required to provide a rationale for new treatment strategies.

MET is the most widely prescribed oral hypoglycaemic medication for type 2 diabetes worldwide. Emerging evidence indicates that MET has beneficial effects on health beyond those associated with amelioration in glycaemia [27]. It has been confirmed that the mechanisms of MET are important for targeting fundamental pathways in biological ageing, such as activating AMPK, augmenting autophagy, inhibiting inflammation and exerting anti-oxidative effects [28]. In humans, MET has been used in clinical practice for more than 60 years; it has a high safety profile and is uniquely positioned to intervene in several crucial pathways responsible for ageing and age-related diseases. However, the effects of MET on ARC and the regulatory mechanism of those effects have never been reported. Consequently, in our study, chronic low-dose MET administration in naturally aged mice improved the transparency of the lens (Fig. 2a) and attenuate the senescence of LECs in vivo (Fig. 2).

It is widely acknowledged that MET activates the AMPK pathway that is the potential mechanism of MET to exert an anti-ageing effect [29]. AMPK, a cellular energy sensor, plays a crucial role in regulating cellular energy balance [30]. Several studies have illustrated that AMPK serves as an integrator and mediator of several pathways and processes associating energetics to longevity. In our study, we found that the inactivation of AMPK was a feature of senescent LECs in naturally aged mice (Fig. 3). Subsequently, MET was shown to stimulate the AMPK pathway to prevent the senescence of LECs (Fig. 3). As an established AMPK activator, the role of MET in the prevention of ageing is generally attributed to its effects on modulating downstream signalling pathways, such as restoration of autophagy, activation of Sirt1 and Foxo1 and suppression of mTOR [31, 32]. Our study provided a new line of evidence stressing the importance of AMPK function activated by MET on preventing the occurrence of ARC.

As mentioned above, AMPK communicates with numerous pathways and proteins to exert an anti-senescence effect, including the augmentation of autophagy. Autophagy, emerging as a core process for longevity assurance, has attracted widespread interest as a potential therapeutic target for age-related diseases. Autophagy is a fluid, multi-step complex biological process. The complete process of autophagy is called autophagic flux, which is widely used to reflect the level of autophagy. As an indicator of autophagy, LC3-II is tightly bound to the autophagosomal membrane that is degraded by lysosomal enzymes. Similarly, p62, an autophagy-specific substrate, is usually degraded in autophagolysosome, and its expression indirectly reflects the level of autophagy. In the present study, our findings implied that the level of autophagy was distinctly declined in senescent LECs (Fig. 4), whereas MET reversed the age-related impairment of autophagy (Fig. 4). The anti-ageing molecular mechanism of MET is attributed to the primary function of AMPK in cells because it generally enhances autophagy via AMPK activation [33]. Moreover, MET is shown to augment autophagy directly, thereby contributing to the prevention of senescence [34]. However, more detailed studies are needed to elucidate the underlying mechanisms, proving that MET augments autophagy via an independent or a dependent AMPK pathway.

It has been demonstrated that the prevalence of ARC was not associated with sex in populations having an Asian origin [35, 36]. Therefore, we did not consider sex as a factor that may affect ARC. Our study should be interpreted considering the above-mentioned limitations.

## Conclusion

In summary, we proved that inactivation of the AMPK pathway and age-related impairment of autophagy might contribute to the senescence of LECs and the occurrence of ARC. In addition, the data from our experiments revealed that MET effectively delayed the senescence of LECs and the formation of ARC via activation of the AMPK pathway and augmentation of autophagy. Our findings are a basic line for further investigation of the molecular mechanisms that are useful in the role of MET during the ageing process of ARC and will be informative for more intensive studies to the development of new strategies against the occurrence and development of ARC.

## Materials and methods

### Reagents

MET was purchased from Bristol-Myers Squibb Company (China). Antibodies against p21 (ab188224), p53 (ab131442), phosphorylated AMPKα (p-AMPK) (Thr172) (ab133448), AMPKa1 (ab32047), β-actin (ab8226) and SQSTM1/p62 (3340-1) were obtained from Abcam (MA, USA). Antibodies against LC3 (12741) and phospho-acetyl CoA carboxylase (p-ACC) (Ser79) (3661) were purchased from Cell Signalling Technology (MA, USA).

### Animals and treatment

Male C57BL6J mice that were 3 and 8 months old were purchased from Beijing Vital River Laboratory Animal Technology Co., Ltd. (Beijing, China). The mice were housed in temperature-controlled (temperature, 20–25 °C and relative humidity, 55 ± 15%) conditions with a 12 h light/dark cycle, with free access to food and water. All procedures and protocols conducted on animals were approved by the ethics committee of the Harbin Medical University.

The mice were divided into the following three groups: (i) control group having young mice (3 months old, bodyweight = 29.72 ± 1.66 g, *n* = 40) received a standard purified mouse diet and water ad libitum; When the mice were 10 months old, 120 mice were randomly divided into two groups: naturally aged group and MET group. (ii) naturally aged group (older mice, 20 months old, bodyweight = 32.98 ± 1.93 g, *n* = 60) received a standard purified mouse diet and water ad libitum; (iii) MET group (20 months old, bodyweight = 31.21 ± 2.81 g, *n* = 60) was maintained on a standard purified mouse diet and water until the mice reached 10 months of age; after which, the treatments were initiated. Mice in the MET group were fed a diet supplemented with 0.1% MET (Bristol Myers Squibb, China) and water freely for 10 months.

### Isolation of lenses and lens capsules

The mice were euthanised via cervical dislocation. The whole eye was excised immediately from all experimental animals and placed in sterile saline. The optic nerve was placed upwards and fixed with tweezers. An incision was then made on the optic nerve that enters the eye, and the sclera was pulled back to expose the lens. Subsequently, we used tweezers to gently remove the ciliary body fragments attached to the equatorial plane of the lens. Photos of the fresh lens were immediately taken, and the lens was quickly fixed with 4% paraformaldehyde at room temperature. The entire lens capsules were quickly peeled from the remaining lens, snap-frozen in liquid nitrogen and stored at 80 °C for subsequent experiments.

### Haematoxylin and eosin (H&E)

The fresh lens was immediately fixed with 4% paraformaldehyde at room temperature and embedded in paraffin. For histological examination, the tissues were cut into 4-μm slices. After deparaffinisation and rehydration, the prepared paraffin sections were stained with haematoxylin (H) solution for 8 min, followed by 5 dips in 1% acid ethanol (1% HCl in 70% ethanol) and rinsing with distilled water. Subsequently, the sections were stained with eosin (E) solution for 3 min, followed by dehydration with graded alcohol and clearing with xylene. Morphological changes were observed under a microscope (Nikon, Eclipse, Japan).

### Senescence-associated β-galactosidase (SA-β-Gal) assay

The senescence-associated β-galactosidase (SA-β-Gal) activity was measured by using an SA-β-Gal staining kit (Sigma, CS0030). According to the manufacturer’s instructions, the tissue sections, after a series of treatments, were washed with phosphate-buffered saline (PBS) for 5 min (thrice) and then incubated in SA-β-gal staining solution (pH 6.0) at 37 °C without CO_2_ for 24 h. The images were captured under a light microscope (Nikon, Eclipse, Japan). The percentage of SA-β-gal-positive cells was estimated as the mean number of positive cells/the mean number of total cells.

### Quantitative real-time polymerase chain reaction (qRT-PCR)

Total RNA from the lens capsules of mice was extracted using the TRIzol reagent. Reverse transcription was performed with the ExScript RT Reagent Kit (Invitrogen). Quantitative reverse transcription polymerase chain reaction (qRT-PCR) analysis was performed using a SYBR Green Supermix kit (Takara, Tokyo, Japan), with β-actin as an endogenous control. The transcriptions were investigated for several target genes, including FAS (forward, 5´-TTGCTGGCACTACAGAATGC-3´ and reverse, 5´-AACAGCCTCAGAGCGACAAT-3´), p62 (forward, 5´-GGCGCACTACCGCGATGAGGA-3´ and reverse, 5´-TGTTCCCGCCGGCACTCCTT-3´) and β-actin (forward, 5´-TCGTGGGCCGCCCTAGGCAC-3´ and reverse, 5´-TGGCCTTAGGGTTCAGGGGGG-3´. The relative levels of each gene expression were normalised to β-actin and calculated using the 2^−△△CT^ method.

### Immunohistochemical (IHC) staining

A total of 5 representative sections from each of the 10 selected lenses per group were used for immunohistochemical (IHC) analysis to assess the expression of p21, p53, p-AMPK, p-ACC, LC3 and p62. After the prepared samples were incubated at 60 °C for 2 h, they were passed through xylene and graded alcohol series for deparaffinisation. Subsequently, the samples were boiled in 0.1 M of citric acid (pH 6.1) for 30 min and cooled to room temperature. After washing three times with PBS, the samples were soaked with 0.3% H_2_O_2_ to inhibit endogenous peroxidase activity. Subsequently, primary antibodies were added and cooled at 4 °C, overnight. The corresponding secondary antibodies were incubated for 1 h at 37 °C. After washing with PBS, the samples were incubated in diaminobenzidine (DAB) until the desired stain intensity developed. Images of the immunostained cells were captured using the Nikon Eclipse microscope (Nikon, Eclipse, Japan).

### Western blot analysis

Total protein from lens capsules of mice was extracted using radioimmunoprecipitation assay (RIPA) buffer with protease inhibitor cocktail (Pierce, USA), and a bicinchoninic acid (BCA) kit (Thermo Fisher Scientific, USA) was used to quantify the protein concentration. The total protein (40 μg) was analysed using sodium dodecyl sulfate-polyacrylamide gel electrophoresis (SDS-PAGE) and electrophoretically transferred onto polyvinylidene difluoride filter (PVDF) membranes (Millipore, Germany). The membranes were incubated overnight with a primary antibody at 4 °C. The following day, the membranes were incubated with corresponding secondary antibodies for 1 h at 37 °C. The immunoreactive bands were visualised using the chemiluminescent substrate method with a Super Signal West Pico kit (Pierce Biotechnology, USA). Three independent experiments were performed.

### Statistical analysis

The data were expressed as the mean ± standard deviation (SD). Statistical analyses were conducted using the GraphPad Prism 7.0 software and Microsoft Excel. Protein was quantified using the Image J software. The Student’s *t*-test and analysis of variance (ANOVA) were used to calculate the statistical significance of the experimental data. Differences with a *P‑*value <0.05 were considered statistically significant. All experiments were repeated independently at least three times.

## Data Availability

The datasets used and analysed during the current study are included in this published article.
